# Pharmaceutical Potential of Synthetic and Natural Pyrrolomycins

**DOI:** 10.3390/molecules201219797

**Published:** 2015-12-04

**Authors:** Stella Cascioferro, Maria Valeria Raimondi, Maria Grazia Cusimano, Demetrio Raffa, Benedetta Maggio, Giuseppe Daidone, Domenico Schillaci

**Affiliations:** 1 Dipartimento di Scienze e Tecnologie Biologiche, Chimiche e Farmaceutiche—Sezione di Chimica e Tecnologie Farmaceutiche—Università degli Studi di Palermo, Via Archirafi 32, Palermo 90123, Italy; mariavaleria.raimondi@unipa.it (M.V.R.); demetrio.raffa@unipa.it (D.R.); benedetta.maggio@unipa.it (B.M.); giuseppe.daidone@unipa.it (G.D.); domenico.schillaci@unipa.it (D.S.); 2 IEMEST, Istituto Euromediterraneo di Scienza e Tecnologia, Via Emerico Amari, 123, Palermo 90139, Italy

**Keywords:** pyrrolomycins, antibiotic resistance, antibiofilm agents, pyoluteorin, pentabromopseudilin

## Abstract

The emergence of antibiotic resistance is currently considered one of the most important global health problem. The continuous onset of multidrug-resistant Gram-positive and Gram-negative bacterial strains limits the clinical efficacy of most of the marketed antibiotics. Therefore, there is an urgent need for new antibiotics. Pyrrolomycins are a class of biologically active compounds that exhibit a broad spectrum of biological activities, including antibacterial, antifungal, anthelmintic, antiproliferative, insecticidal, and acaricidal activities. In this review we focus on the antibacterial activity and antibiofilm activity of pyrrolomycins against Gram-positive and Gram-negative pathogens. Their efficacy, combined in some cases with a low toxicity, confers to these molecules a great potential for the development of new antimicrobial agents to face the antibiotic crisis.

## 1. Introduction

Antibiotic resistance of common pathogenic microorganisms is a topic of great concern that has finally attracted the attention of mass-media and governments. Drug-resistant bacteria are responsible for some 30,000 deaths per year in the UK and Europe and it is estimated that 23,000 people in the United States die from pathogens that are not susceptible to treatment with any of the current antibiotics [1]. There is therefore an urgent need to introduce novel antimicrobial molecules in therapy, and there are several possible alternative strategies to conventional antibiotics to counteract antibiotic resistance [2,3,4,5,6].

No new chemical classes of broad-spectrum antibiotics and few molecules with narrow spectrum have been found in the recent past [7]. The increase of antimicrobial resistance has stimulated research aimed towards the discovery of inhibitors of new bacterial targets [8,9]. In addition, more than 80% of infections are associated to the growth of a sessile community of pathogenic bacteria, the so-called biofilms, which are intrinsically resistant to conventional antibiotics [10].

Conventional antibiotics are effective against planktonic cells but are usually ineffective against biofilm-associated infections because of the high level multi-factorial resistance provided by biofilm mode of growth [11].

The development of anti-biofilm drugs, or the discovery of antibiotics with this additional property, may contribute to combat the emergence of antibiotic-resistance.

The reconsideration of old molecules that were previously largely replaced by more “modern” antibiotics might represent a rapid strategy for the treatment of drug-resistant infectious diseases [12,13]. Valuable examples are provided by colistin, a polymixin produced by *Paenibacillus polymyxa* subspecies colistinus [14,15], or temocillin, fosfomycin, mecillinam, nitrofurantoin and chloramphenicol for multi drug resistant (MDR) Gram-negative bacteria, and trimethoprim-sulfamethoxazole for methicillin-resistant *Staphylococcus aureus* (MRSA) [16]. Furthermore, some authors have recently suggested the use of tetracyclines for multidrug-resistant *Acinetobacter baumannii* infections [17].

Many antimicrobial molecules have been discovered in the past, but they were never developed for clinical practice. These include natural halogenated pyrroles antibiotics, such as pyoluteorin, discovered in 1958, and the pyrrolomycins, isolated in 1983.

Here, we will comment on the possibility that some of these old molecules are valuable therapeutic agents to defeat the widespread emergence of bacterial resistance, placing emphasis upon their profile of efficacy, safety, and tolerability. We will specifically describe the pyrrolomycins, a class of polyhalogenated pyrrolic compounds, endowed with potent antibacterial activity, particularly against Gram-positive, and, in some cases, Gram-negative pathogens, including *Pseudomonas aeruginosa*, and showing the ability to target biofilms.

## 2. Halogenated Pyrroles Related to Pyrrolomycins

**Figure 1:**
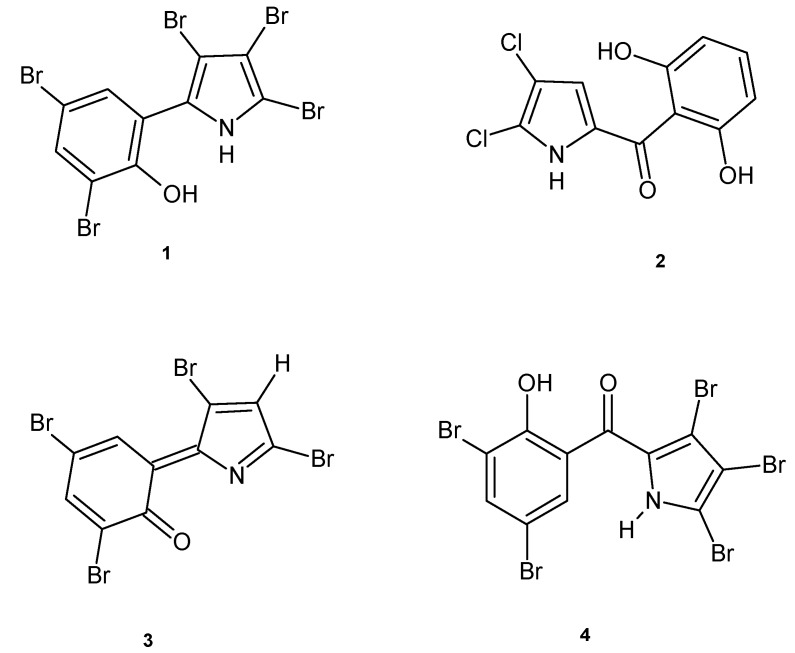
Structures of compounds **1**–**4**.

The antibacterial properties of halogenated pyrroles, such as pentabromopseudilin (**1**), isolated from the marine bacterium *Alteromonas luteoviolaceus*, and pyoluteorin (**2**) isolated from *Pseudomonas aeruginosa* (Figure 1), both structurally related to pyrrolomycins, have been known for a long time.

Burkholder *et al.* described in 1966 the antibiotic properties of pentabromopseudilin, (2,3,4-tribromo-5(1′-hydroxy, 2′,4′-dibromophenyl)pyrrole, **1**) [18]. The antibacterial activity of pentabromopseudilin is due to its ability to interfere with the synthesis of macromolecules in Gram-positive and Gram-negative bacteria, probably through the formation of a prototropic isomer by hydrogen shift and elimination of hydrogen bromide. This reaction proceeds with the formation of a quinonoid system **3** (Figure 1) that may bind SH or NH groups of the target proteins.

Although there are different biological activities described for this molecule, such as antifungal and antitumor activity, inhibition of cholesterol biosynthesis, and plant protection [19], here we specifically focus on the antibacterial activity, considering this class of old compounds as a good starting point to thwart the worldwide prevalence of antibiotic–resistant pathogens and the lack of novel antibiotics. The antibacterial activity observed for pentabromopseudilin is reported in Table 1.

**Table 1:** Antibacterial activity of pentabromopseudilin expressed as Minimum Inhibitory Concentration (MIC) or half maximal inhibitory concentration (IC_50_) in μM.

Microorganisms	Pentabromopseudilin (1)	References
*Acinetobacter calcoaceticus*	>90.3 μM	[19]
*Bacillus brevis*	1.81 µM	[19]
*Bacillus subtilis*	1.81 µM	[19]
MRSA (ATCC 43300)	(IC_50_) = 0.181 µM	[20]

The introduction in the pentabromopseudilin of a carbonyl group between the phenyl group and the pyrrole moiety, *i.e*., compound **4** (Figure 1), entails an increase in antibacterial activity against *Escherichia coli* and *Bacillus subtilis*, whereas N-methylation is disadvantageous for the antibacterial activity [19,21].

For pyoluteorin (**2**) activity against *S. aureus*, *P. aeruginosa*, *E. coli* and *Pseudomonas vulgaris* with MIC values of 11.39 µM, 459 µM, 22.79 µM and 459 µM, respectively, is reported [22]. The study of Structure Activity Relationship (SAR) of the 2-aroyl-4,5-dihalopyrroles [22,23] has shown that: (i) the presence of one or more electron-withdrawing groups on the pyrrole is required for the antibacterial activity; (ii) the replacement of chlorine on the pyrrole ring with more electropositive halogens, such as bromine and iodine, produces less active compounds towards *S. aureus* and *E. coli* [22]; (iii) the replacement of chlorine atoms with nitro groups causes an increase of antibacterial activity [23]; (iv) the attendance on the phenyl ring of a 4-CF_3_, 2,5-diOH or 2,4-diOH leads to compounds with better or comparable antibacterial activity with respect to pyoluteorin against *S. aureus*, with MIC values in the range 0.98–3.9 µg/mL, but with only a weak activity on *E. coli* (MIC > 125 µg/mL).

The acidity of the pyrrole NH group appeared to be important for the activity of this class of compounds; accordingly, the presence of a more electronegative group, such as the nitro group, which causes a significant increase in NH acidity, is associated with an improvement of microbiological activity, probably because it facilitates a stronger binding to the active site of the receptor target [23].

**Figure 2:**
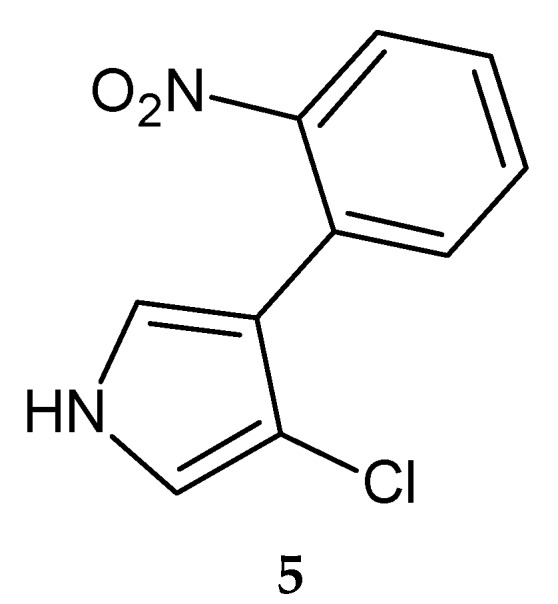
Structure of pyrrolnitrin **5**.

Another natural halogenated pyrrole derivative showing antimicrobic actity is the antibiotic pyrrolnitrin (**5**) (Figure 2), produced by *Pseudomonas* spp. [24,25,26], *Myxococcus fulvus* [27], and *Enterobacter agglomerans* [28]. It shows antibacterial activity against Gram-positive pathogens, in particular against certain *Streptomyces* species, such as *S. antibioticus* and *S. violaceoruber* (MIC = 0.89 μM), against fungal strains, such as *Paecilomyces variotii* and *Penicillium puberulum* (MIC in the range 8.9–26.9 µM) [29], and against a range of mycobacteria, including *Mycobacterium tuberculosis* (MIC in the range 17.9–35.9 µM) [30]. This compound was launched in 1966 as an antifungal OTC drug in Japan.

Studies on the mechanism of action of pyrrolnitrin showed that it probably blocks electron transfer between the dehydrogenases and the cytochrome components of the respiratory chain [29].

## 3. Natural Pyrrolomycins

**Figure 3:**
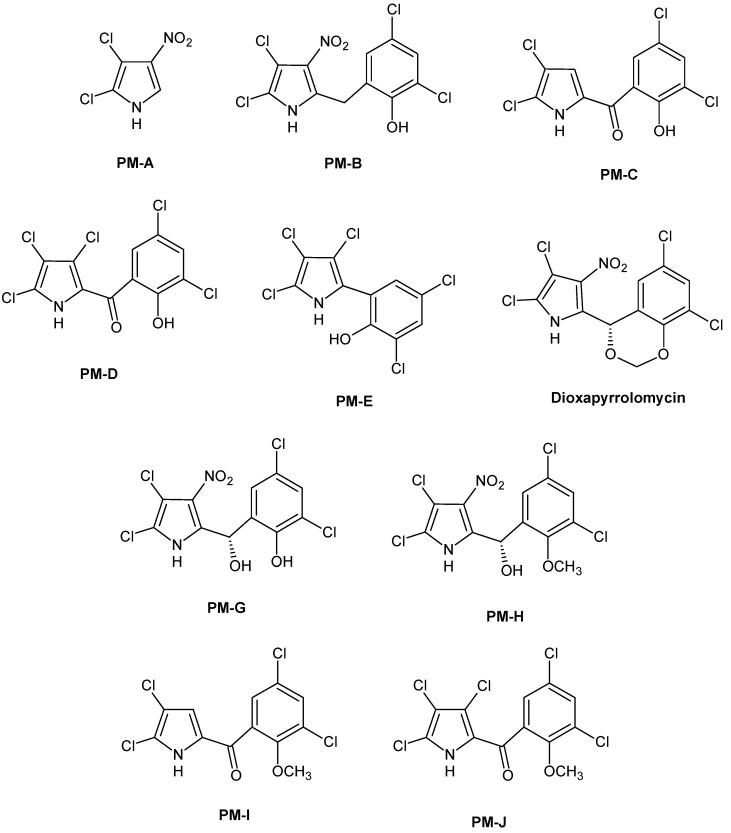
Structures of pyrrolomycins A, B, C, D, E, G, H, I, J and dioxapyrrolomycin.

Pyrrolomycins (PM) are polyhalogenated metabolites known for their potent antimicrobial properties [31], isolated from the fermentation broth of *Actinosporangium* and *Streptomyces* *species* [32,33,34,35,36,37]. The antibiotics of this family include pyrrolomycins A, B, C, D, E, G, H, I, J and dioxapyrrolomycin (Figure 3).

Important structural features of the pyrrolomycins are: (i) their high halogenation degree, which is usually represented by chlorine substituents; and (ii) in some cases, the presence of a nitro group at the position 3 of the pyrrole ring. The antibacterial activity of these derivatives may be closely related to their halogen content, because it increases as the degree of halogenation [38,39].

### Antibacterial Activity of Natural Pyrrolomycins

Pyrrolomycins A and B are active against Gram-positive pathogens, such as *S. aureus*, *S. epidermidis*, *S. faecalis*, and *Bacillus anthracis* and Gram-negative bacteria, such as *E. coli*, *Salmonella typhi*, *Klebsiella pneunioniae* and *Shigella sonnei* with MIC values in the range of 0.55–69.1 µM for PM-A, and in the range of 0.28–35.11 µM for PM-B. The antibacterial activity of both pyrrolomycins A and B is comparable, although pyrrolomycin A is more active against Gram-negative bacteria and pyrrolomycin B against Gram-positive bacteria.

Pyrrolomycins C, D and E are active against Gram-positive pathogens such as *S. aureus*, *S. faecalis* and *Bacillus anthracis*, as reported in Table 2. Pyrrolomycins C and E are inactive against Gram-negative pathogens, such as *E. coli*, *Salmonella typhi*, *Klebsiella pneunioniae* and *Shigella sonnei*, whereas pyrrolomycin D showed a moderate activity with MIC values ranging from 4.34 to 34.78 µM.

Pyrrolomycins C and D are similar to pyoluteorin produced by *Pseudomonas*, but the better activity of pyrrolomycin D against Gram-positive bacteria is probably due to the introduction of a chlorine atom into pyrrole moiety. Pyrrolomycin D was the most active compound among the natural pyrrolomycins, being even more active than vancomycin when tested against *S. aureus*, *S. epidermidis*, *E. faecalis*, *S agalactiae*, *L. monocytogenes*, and *B. subtilis* with MIC values, in most of cases ≤0.002 µM [40].

Dioxapyrrolomycin is primarily active against Gram-positive bacteria, as demonstrated by the MIC values in the range of 0.077–0.64 µM for *S. aureus* and *S. faecalis*. The chirality and novel 1,3-dioxane ring may confer additional unique biological properties to this molecule in comparison to other members of the pyrrolomycin class [31]. The antibacterial activities of natural pyrrolomycins are summarized in Table 2.

**Table 2:** Antibacterial activities of natural pyrrolomycins.

Organism	MIC (µM)					
	PM-A	PM-B	PM-C	PM-D	PM-E	Dioxapyrrolomycin
*Staphylococcus aureus* 209P JC-1	17.29	0.56	0.61	≤0.069	5.07	0.077~0.64
*Streptococcus faecalis* ATCC 8043	34.53	1.09	1.2	≤0.069	5.07	0.644
*Bacillus anthracis*	8.62	0.28	0.31	≤0.069	≤0.16	-
*Escherichia coli*	34.53	35.11	>307.72	17.39	>325.18	>329.87
*Citrobacter freundii* GN 346	34.53	35.11	>307.72	17.39	>325.18	>329.87
*Salmonella typhi* 0-901-W	17.29	35.11	>307.72	17.39	>325.18	-
*Shigella sonnei* EW 33 Type I	34.53	35.11	>307.72	34.78	>325.18	-
*Klebsiella pneumoniae* PCI 602	17.29	35.11	>307.72	17.39	>325.18	>329.87
*Proteus vulgaris* OX-19	34.53	35.11	>307.72	4.34	20.32	-
*Serratia marcescens* MB-3848	34.53	70.23	>307.72	34.78	>325.18	>329.87
*Pseudomonas aeruginosa* MB-3829	69.07	35.11	>307.72	69.56	>325.18	>329.87
*Cryptococcus neoformans* Cr-1	138.14	280.91	>307.72	17.39	>325.18	-
*Candida albicans*	552.55	280.91	>307.72	278.23	>325.18	>1319.48
*Aspergillus fumigatus*	138.14	280.91	>307.72	278.23	>325.18	>1319.48

The pyrrolomycins G-H-I-J were obtained from cultures of *Streptomyces fumanus* [36]. Pyrrolomycin G and pyrrolomycin H, together with dioxapyrrolomycin, are the only pyrrolomycins that possess a chiral center. The crystal structure the *N*-methyl derivative of dioxapyrrolomycin revealed an S absolute stereochemistry.

The pyrrolomycins G, H and J were tested against Gram-positive, *S. aureus* WT and MRSA, and the Gram-negative bacteria *E. faecium* VR, *E. coli* WT, and *E. coli* imp. The activities are summarized as MIC values in Table 3 [41].

**Table 3:** Antibacterial activities of pyrrolomycins G, H and J.

Organism	MIC (µM)		
	PM-G	PM-H	PM-J
*S. aureus* WT	21	2.6	2.6
*S. aureus* MRSA	21	5.1	5.3
*E. faecium* VR	21	NT	NT
*E. coli* WT	>300	>300	>300
*E. coli* imp	86	10.3	21.4

NT = not tested.

**Figure 4:**
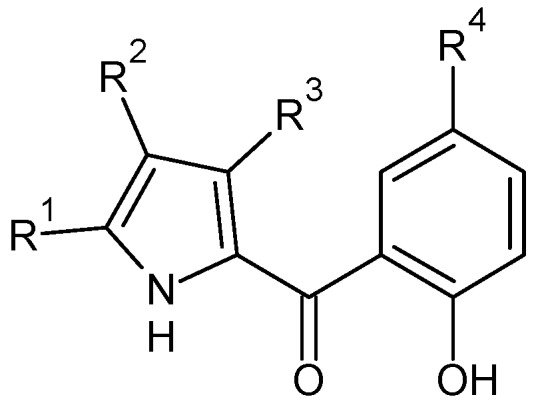
Chemical structures of pyrrolomycins F.

Pyrrolomycins F1, F2a, F2b and F3 (Figure 4) were isolated from *Actinosporangium vitaminophilum* sp. nov. when bromide ions were added to the culture medium [42].

Pyrrolomycins F showed strong activities against Gram-positive bacteria including *S. aureus*, *S. epidermidis* [39], *Streptococcus faecalis*, and *Bacillus anthracis*, and only moderate activities against Gram-negative bacteria like *E. coli*, as shown in Table 4.

**Table:** 

Pyrrolomycin	R^1^	R^2^	R^3^	R^4^
F1	Br	Br	Br	Br
F2a	Br	Cl	Br	Br
F2b	Cl	Br	Br	Br
F3	Cl	Cl	Br	Br

**Table 4:** Antibacterial activities of PM-F reported as MIC (µM).

Microorganisms	MIC (µM)				Reference
	F1	F2a	F2b	F3	
*S. aureus* ATCC 29213	≤0.0019	≤0.0019	≤0.0019	≤0.0019	[40]
*S. aureus* ATCC 25923	0.04	0.04	0.04	0.04	[40]
*S. epidermidis* DSM 3269	0.012	0.012	0.012	0.012	[40]
*S. epidermidis* RP62A	0.004	0.004	0.004	0.004	[40]
*Staphylococcus aureus* 209P JC-1	≤0.002	≤0.002	≤0.002	≤0.002	[39]
*Streptococcus faecalis* ATCC 8043	0.004	0.004	0.004	0.004	[39]
*Bacillus anthracis*	<0.099	<0.099	<0.099	<0.099	[39]
*Escherichia coli*	<0.099	<0.099	<0.099	<0.099	[39]
*Citrobacter freundii* GN 346	<0.099	<0.099	<0.099	<0.099	[39]
*Salmonella typhi* 0-901-W	12.43	12.43	12.43	12.43	[39]
*Shigella sonnei* EW 33 Type I	12.43	12.43	12.43	12.43	[39]
*Klebsiella pneumoniae* PCI 602	12.43	12.43	12.43	12.43	[39]
*Proteus vulgaris* OX-19	12.43	12.43	12.43	12.43	[39]
*Serratia marcescens* MB-3848	12.43	12.43	12.43	12.43	[39]
*Pseudomonas aeruginosa* MB-3829	6.22	6.22	6.22	6.22	[39]
*Cryptococcus neoformans* Cr-1	6.22	6.22	6.22	6.22	[39]

Pyrrolomycins C, D, F1, F2a, F2b and F3 were also tested for their anti-staphylococcal biofilm activity. With the exception of pyrrolomycin C, all tested compounds were active against all staphylococcal biofilms at the concentration of 1.5 µg/mL (4.17 µM, 2.98 µM, 3.27 µM, and 3.62 µM for pyrrolomycin D, F1, F2a,b, and F3, respectively), the inhibition percentages being >60% for all compounds, and, in many cases, >80%. At the lower concentration of 0.045 µg/mL, pyrrolomycin F3 (0.11 µM) showed the best activity with an inhibition percentage >50% against all tested strains and the remarkable selectivity index (ratio between IC_50_ on human cell and antibiofilm concentration) of 1333 [39].

Toxicity studies *in vivo* have been carried out on JCL-ICR mice for dioxapyrrolomycin, which unfortunately was very toxic with an oral LD_50_ of 13 mg/kg [31]. The other natural pyrrolomycins were less toxic: pyrrolomycin A and B showed LD_50_ values of 21.2 and about 100 mg/kg, respectively, when administered i.p. to JCL-ICR mice [37]; pyrrolomycins C and D showed LD_50_ values of 50 mg/kg and 20 mg/kg, respectively [35].

## 4. Synthetic Pyrrolomycins

Pentatomic nitrogenous heterocyclic compounds are widely described in the literature for a variety of biological activities [43,44,45] including the antibacterial activity [46,47,48]. This observation, combined with the interesting biological results observed with the natural pyrrolomycins, encouraged the synthesis of new analogs.

In 1973, Bailey *et al.* synthesized a series of compounds related to pyoluterin via three different synthetic routes: (i) acylation of pyrrole with the Grignard reagent; (ii) acylation with 4,5-dihalopyrrol-2-carbonyl chloride; and, (iii) base-catalyzed condensation of pyrrole with arylaldehydes. Compounds **6a**–**j** showed good *in vitro* antibacterial activities against a variety of pathogens (Table 5), but none was able to prevent mortality in mice infected with *S. aureus* or *Klebsiella pneumoniae* [22].

**Table 5:** *In vitro* antibacterial activity of compounds **6a**–**j**. 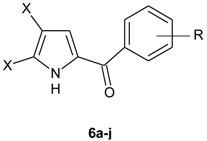

Compound	X	R	MIC µM			
			*S. aureus*	*P. aeruginosa*	*E. coli*	*P. vulgaris*
**6a**	Cl	2,6-(OH)_2_	11.39	459.42	22.78	459.42
**6b**	Br	2-Cl	42.92	>343.94	343.94	>343.94
**6c**	Cl	3-Cl	28.41	227.66	>227.66	>227.66
**6d**	Cl	4-F	60.45	484.33	>484.33	>484.33
**6e**	Br	4-F	44.96	360.25	>360.25	>360.25
**6f**	Cl	4-CF_3_	10.06	>405.74	>405.74	>405.74
**6g**	Br	2,4,6-(CH_3_)_3_	42.04	>336.86	>336.86	>336.86
**6h**	Cl	2,5-(OH)_2_	3.6	459.42	459.42	229.71
**6i**	Br	2,5-(OH)_2_	43.21	346.27	346.27	173.13
**6j**	Cl	2,4-(OH)_2_	14.33	>459.42	115.04	>459.42

**Figure 5:**
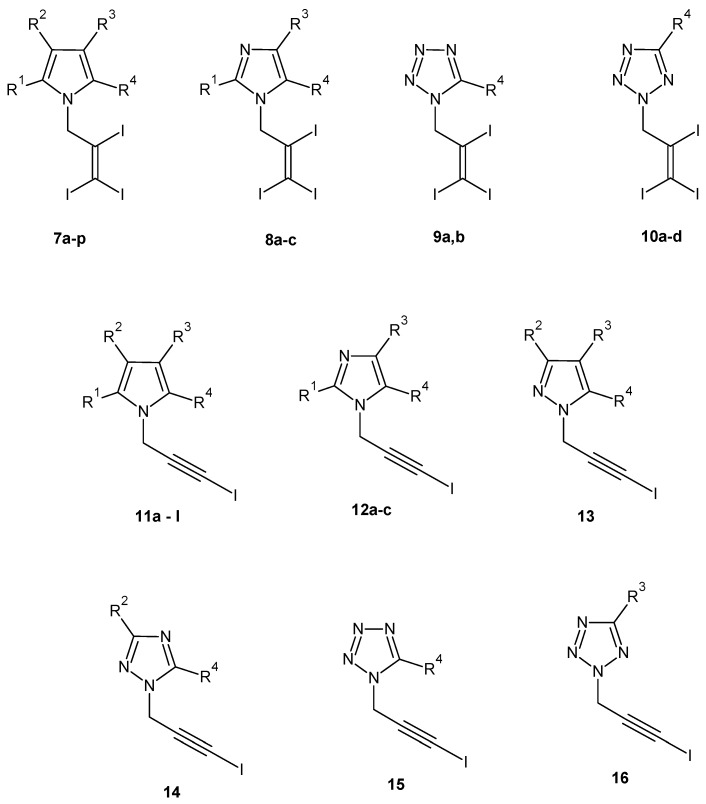
Structures of compounds **7**–**16**.

Koyama *et al.* submitted a patent on triiodoallyl- or iodopropargyl-substituted heterocyclic aromatic compounds endowed with remarkable antibacterial and antifungal activities. The inventors changed the structure of pyrrolomycin A by introducing a triiodoallyl group (compounds **7a**–**p**, **8a**–**c**, **9a**,**b**, **10a**–**d**) or an iodopropargyl group (compounds **11a**–**l**, **12a**–**c**, **13**, **14**, **15**, **16**) to the nitrogen atom of the pyrrole ring in an attempt to obtain compounds more potent than pyrrolomycin A (Figure 5, Table 6). The new structures have been synthesized by reaction of an unsubstituted or substituted nitrogen-containing heterocyclic compound with a reactive derivative of a 2,3,3-triiodoallyl alcohol or a reactive derivative of a 3-iodopropargyl alcohol in the presence of a base in an inert organic solvent. All new compounds showed a good biological activity against various bacterial and fungi strains, with MICs ranging from 0.09 µg/mL to >50 µg/mL [49].

**Table 6:** Substituents R^1^, R^2^, R^3^ and R^4^ of compounds **7**–**16**.

Compound	R^1^	R^2^	R^3^	R^4^
**7a, 11a**	Cl	Cl	NO_2_	H
**7b, 11b**	Cl	H	NO_2_	H
**7c**	H	H	NO_2_	H
**7d**	Cl	Cl	COOC_2_H_5_	H
**7e**	Cl	Cl	COOCH_3_	H
**7f**	Cl	H	COOC_2_H_5_	H
**7g**	H	COOC_2_H_5_	H	H
**7h**	H	COOCH_3_	H	H
**7i**	H	Cl	3-Cl-2-NO_2_-C_6_H_5_	H
**7j**	H	3-Cl-C_6_H_5_	H	H
**7k, 11i**	H	C_6_H_5_	H	H
**7l**	Cl	Cl	NO_2_	Cl
**7m, 11j**	Cl	Cl	H	COOCH_3_
**7n**	COOCH_3_	H	Cl	H
**7o, 11k**	COOCH_3_	H	H	H
**7p, 11l**	NO_2_	H	H	H
**8a, 12a**	H	-	H	H
**8b, 12b**	NO_2_	-	H	H
**8c, 12c**	H	-	NO_2_	H
**9a, 15**	-	-	-	H
**9b**	-	-	-	CH_3_
**10a, 16**	-	-	-	H
**10b**	-	-	-	CH_3_
**10c**	-	-	-	C_6_H_5_
**10d**	-	-	-	NHCOCH_3_
**11c**	H	NO_2_	H	H
**11d**	Cl	Cl	H	Cl
**11e**	Br	Br	NO_2_	H
**11f**	Cl	Cl	COOC_2_H_5_	H
**11g**	H	COOC_2_H_5_	H	H
**11h**	H	Cl	2-NO_2_-3-Cl-C_6_H_5_	H
**13**	H	H	H	H
**14**	H	H	H	H

**Figure 6:**
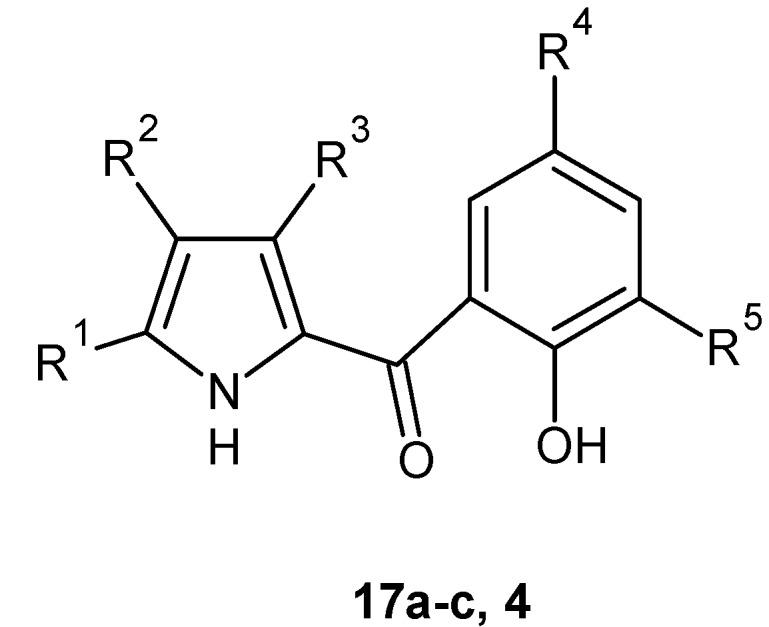
Structures of derivatives **17a**–**c** and **4**.

Schillaci *et al.* [50] reported the synthesis of bromo analogs of pyrrolomycins **17a**–**c** and **4** (Figure 6) by reaction of pyrrolylmagnesium bromide with 2-methoxybenzoyl chloride and subsequent demethylation of the methoxy group using anhydrous aluminum chloride to obtain the (2-hydroxyphenyl)(1*H*-pyrrol-2-yl)methanone. Finally, the bromination was carried out with cupric(II) bromide in organic solvent. Compounds **17c** and **4** showed a remarkable antibacterial activity against *S. aureus* ATCC 25,923 (MIC values = 0.099 and 0.017 µM, respectively); in particular, compound **4** was about 200 times more potent than the aminoglycoside, amikacin, against *S. aureus* [50]. The encouraging results led the authors to test compounds **17a**–**c** against ten clinical *S. aureus* strains, five susceptible and five resistant to methicillin [51]. Results, expressed as MIC values, showed that the antibacterial activity was correlated to the number of bromine atoms present on the molecule and the most active compound was the pentabromo analog **4** (Table 7). The authors continued the studies by testing, *in vitro*, compound **4** on seven reference Gram-positive bacterial strains including *S. epidermidis*, *S. aureus*, *Listeria monocytogenes*, *Streptococcus agalactiae*, *Bacillus subtilis*, and *Enterococcus faecalis* [38]. The activity of compound **4** was compared to the activity of vancomycin, and MIC and MBC values were determined. Compound **4** was found to be active against all tested strains with better MIC (ranging from 0.003 to 0.016 µM) and MBC (ranging from 0.63 to 21.4 µM) values than vancomycin, which showed MIC values ranging from 0.69 to 2.76 µM, and MBC values from 1.38 to >5.5 µM.

**Table:** 

Compound	R^1^	R^2^	R^3^	R^4^	R^5^
**17a**	Br	Br	H	H	H
**17b**	Br	Br	H	Br	H
**17c**	Br	Br	H	Br	Br
**4**	Br	Br	Br	Br	Br

**Table 7:** Minimum inhibitory concentration (MIC) in µM against clinical *S. aureus* strain susceptible or resistant to methicillin (s: methicillin susceptible strains; r: methicillin resistant strains).

*S. aureus*	(MIC) in µM			
Strain	17a	17b	17c	4
1s	17	0.87	0.35	0.06
2s	17	0.87	0.17	0.06
3s	34.7	3.53	0.73	0.01
4s	17	0.87	0.17	0.06
5s	17	3.53	0.35	0.09
1r	8.69	0.87	0.07	0.01
2r	17	0.21	0.07	0.01
3r	17	0.42	0.17	0.03
4r	17	1.76	0.17	0.01
5r	8.69	3.53	0.35	0.06

In addition, compound **4** was also found to be active against preformed *S. epidermidis* and *S. aureus* biofilms with inhibition percentages ranging from 100 to 49.4 and 89 to 58, respectively, at concentrations of 0.08–2.5 µM.

In 2006, the Authors synthesized several 2-(2′-hydroxybenzoyl)pyrrole bromine derivatives (compounds **18a**,**b** and **19a**,**b**) to investigate whether the introduction of a keto or methylene spacer between the phenol and pyrroloyl moiety led to more active compounds [52]. Compounds **18a**,**b** have been synthesized by reaction of the (3,5-dibromo-2-methoxyphenyl)acetyl chloride with pyrrolylmagnesium bromide in anhydrous diethyl ether to obtain the 2-(3,5-dibromo-2-methoxyphenyl)-1-(1*H*-pyrrol-2-yl)ethanone. Bromination with N-bromosuccinimide and the demethylation with boron tribromide in dry dichloromethane led to compounds **18a**,**b**. Compounds **19a**,**b** were obtained by oxidation with selenium dioxide of the methylene group of the intermediates 1-(4,5-dibromo-3-*R*-1*H*-pyrrol-2-yl)-2-(3,5-dibromo-2-methoxyphenyl)ethanones and subsequent demethylation using anhydrous aluminum chloride in dry dichloromethane. The structural changes generated compounds with less antimicrobial activity with respect to compound **4**; nevertheless, compounds **18a**,**b** and **19a**,**b** resulted more active on *S. aureus* than the comparator amikacin, as shown in Table 8.

**Table 8:** Antimicrobial activity *in vitro*, MIC values expressed in µM for all strains tested. 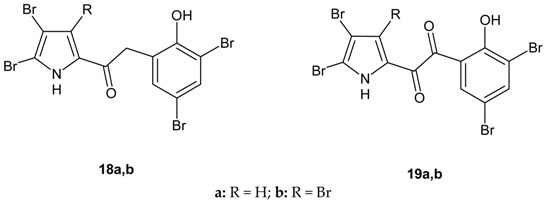

Compound	*S. aureus* ATCC 25923	*E. coli* ATCC 25922	*C. albicans* ATCC 10231
**4**	0.008	10.6	>21.4
**18a**	1.45	>24.1	>24.1
**18b**	0.15	10.4	>20.9
**19a**	2.8	>23.5	>23.5
**19b**	0.6	>20.5	>20.5
Amikacin	1.7	17	n.t.
Amphotericin B	n.t.	n.t.	0.16

**Figure 7:**
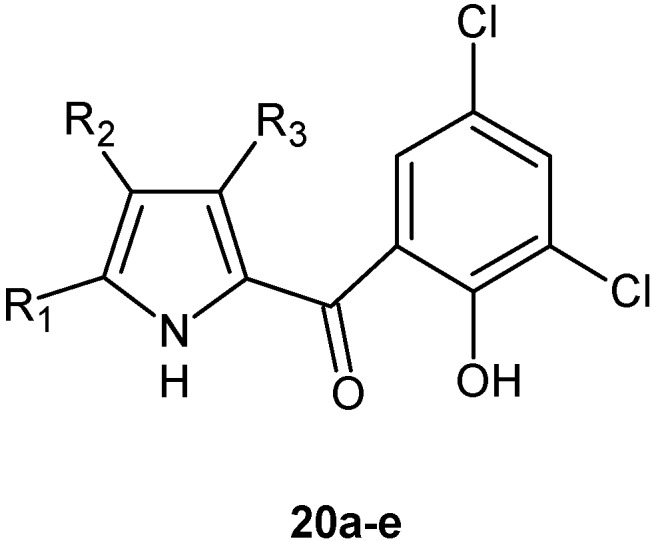
Structures of compounds **20a**–**e**.

Moreover, the Authors reported the synthesis of new halogenated pyrroles related to pyrrolomycins F **20a**–**e** to investigate their anti-Gram-positive and anti-staphylococcal activities and their ability to inhibit the formation of biofilms (Figure 7) [39,40,53].

**Table:** 

Compound	R_1_	R_2_	R_3_
**20a**	Br	Br	Br
**20b**	Br	Cl	Br
**20c**	Cl	Br	Br
**20d**	Cl	Cl	Br
**20e**	Cl	Br	Cl

Compounds **20a**–**e** were synthesized by reaction of pyrrolylmagnesium bromide with the 3,5-dichloro-2-methoxybenzoyl chloride. The introduction of halogen atoms on the pyrrole moiety was conducted by addition of the appropriate equivalents of *N*-bromosuccinimide or *N*-chlorosuccinimide in acetonitrile. Finally, the demethylation using anhydrous aluminum chloride in dry dichloromethane led to compounds **20a**–**e** [53]. Results showed that the replacement of the 4′-bromo atom on the phenolic ring of pyrrolomycins F with two chloro atoms at the 3′ and 5′ position resulted in an increase of antibacterial activity against all strains tested, with MIC values ranging from ≤0.001 to 12.5 µg/mL and MBC values ranging from ≤0.39 µg/mL to 12.5 µg/mL.

Derivatives **20a** and **20d** were particularly interesting, showing MIC values against *S. aureus*, *S. epidermidis*, *E. faecalis*, *S. agalactiae*, *L. monocytogenes* and *B. subtilis* in the range 0.012–0.2 µM and 0.007–0.2 µM respectively. They were more potent than vancomycin, which showed MIC values ranging from 0.6 to 2.7 µM.

Compounds **4**, **20a** and **20d** were also compared to some natural pyrrolomycins for their antistaphylococcal biofilm activity. Compound **20a** showed a substantial antibiofilm activity at 0.09 µM with an inhibition percentage >50% against all tested staphylococcal strains. The activity of compound **20a** was comparable to that exhibited by pyrrolomycin F3 and greater than that exhibited by pyrrolomycins C, D, F1, F2a and F2b. Interestingly, compound **20a** had a selectivity index of 1666, and, therefore, it showed a slightly higher selectivity than pyrrolomycin F3 (selectivity index = 1333). These data are particularly relevant because they refer to the antibiofilm activity. The selectivity indexes of compound **20a** and pyrrolomycin F3 referred to their activities against the planktonic mode of growth were 7500 and 9600, respectively. To our knowledge, synthetic analogues of pyrrolomicins have never been tested for their toxicity in animal models.

The antibiofilm activity of pyrrolomycins against *S. aureus* was also evaluated in terms of log reductions and their efficacy was compared to the efficacy of rifampicin, an antibiotic that is currently used in the clinic in the treatment of staphylococcal biofilm. [54] By using this different biofilm growth model, compound **20a** and pyrrolomycin D were more active than rifampicin [39].

## 5. Conclusions

Resistant pathogens have become a serious threat worldwide and the use of new effective agents against drug-resistant bacteria is an urgent medical need. Such an emergence could be faced by revisiting old antibiotics and carefully monitoring their profile of efficacy, safety, and tolerability. Pyrrolomycins and other molecules are extremely interesting in terms of activity, *in vitro* and *in vivo* toxicity, and additional features including the ability to defeat intrinsic forms of resistance, such as biofilms [39]. Moreover, natural pyrrolomycin F3 and the synthetic analogue **20a** have a very low cytotoxicity in human primary cell cultures, with a selectivity index >1000. This is remarkable if one considers that a good safety margin for a compound to be considered as a potential candidate for clinical development is a selectivity index >200 [55]. Another advantage of pyrrolomycins is related to their simple chemical synthesis, which allows us to easily obtain them although producing organisms are no longer available. It is concluded that pyrrolomycins and their synthetic derivatives are potential compounds for developing novel effective chemical countermeasures against pathogens. These molecules might represent the launching platform for the development of new antimicrobial agents. Data on the efficacy and safety of pyrrolomycins in animal models are urgently needed to determine whether these molecules deserve further consideration.
